# *catena*-Poly[[bis­(thio­cyanato-κ*N*)cobalt(II)]-di-μ-thio­urea-κ^4^ *S*:*S*]

**DOI:** 10.1107/S1600536812033193

**Published:** 2012-07-28

**Authors:** K. Rajarajan, K. Sendil Kumar, V. Ramesh, V. Shihabuddeen, S. Murugavel

**Affiliations:** a Department of Physics, Rajeswari Vedachalam Government Arts College, Chengalpet 603 301, India; b Research and Development Center, Bharathiar University, Coimbatore 641 046, India; c Department of Physics, Thanthai Periyar Government Institute of Technology, Vellore 632 002, India

## Abstract

In the title polymeric complex, [Co(NCS)_2_{SC(NH_2_)_2_}_2_]_*n*_, the asymmetric unit comprises a Co^II^ ion, which is situated on an inversion centre, an *N*-bound thio­cyanate anion and a μ_2_-bridging thio­urea mol­ecule. The Co^II^ atom is coordinated in a distorted octa­hedral fashion within an N_2_S_4_ donor set. The bridging thio­urea ligands link Co^II^ ions into a polymeric chain extending along [100]. The mol­ecular conformation is stabilized by intra­molecular N—H⋯N hydrogen bonds, which generate *S*(6) ring motifs. The crystal packing is stabilized by N—H⋯S inter­actions, which connect the chains into a three-dimensional architecture.

## Related literature

For a general introduction to thio­cyanato complexes, see: Nardelli *et al.* (1957 ▶). For the crystal structure of the analogous Cd^II^ complex, see: Wang *et al.* (2002 ▶). For information on the properties of complexes incorporating these ligands, see: Yuan *et al.* (1997 ▶); Yu *et al.* (2001 ▶); Machura *et al.* (2011 ▶). For the use of Co^II^ complexes with mixed S-donor ligands as precursors to CoS, see: Kropidłowska *et al.* (2008 ▶). For hydrogen-bond motifs, see: Bernstein *et al.* (1995 ▶).
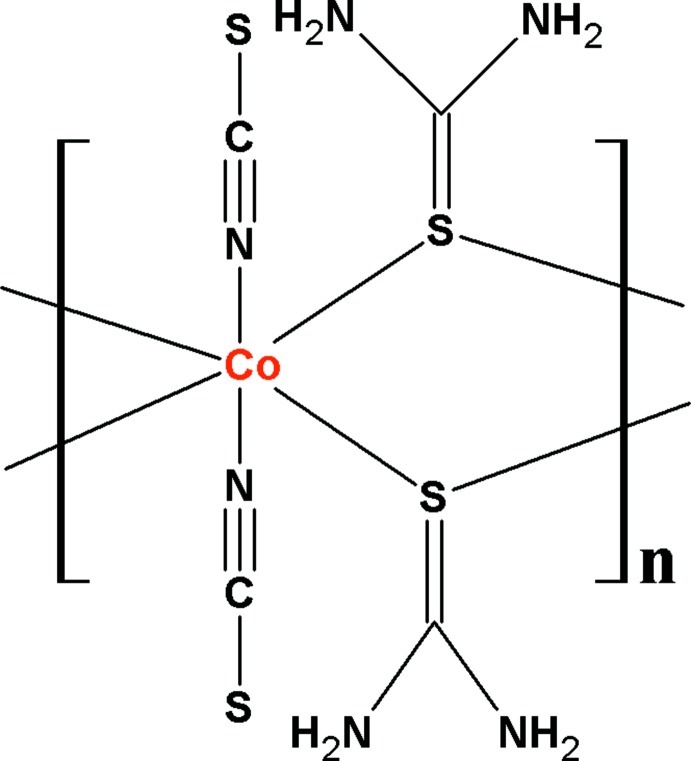


## Experimental

### 

#### Crystal data

[Co(NCS)_2_(CH_4_N_2_S)_2_]
*M* *_r_* = 327.33
Triclinic, 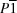

*a* = 3.855 (3) Å
*b* = 7.585 (2) Å
*c* = 10.094 (2) Å
α = 92.424 (3)°
β = 98.172 (2)°
γ = 104.166 (2)°
*V* = 282.4 (2) Å^3^
*Z* = 1
Mo *K*α radiation
μ = 2.23 mm^−1^
*T* = 293 K
0.24 × 0.22 × 0.16 mm

#### Data collection

Bruker APEXII CCD diffractometer
Absorption correction: multi-scan (*SADABS*; Sheldrick, 1996 ▶) *T*_min_ = 0.591, *T*_max_ = 0.699
6452 measured reflections
1844 independent reflections
1764 reflections with *I* > 2σ(*I*)
*R*_int_ = 0.026

#### Refinement

*R*[*F* ^2^ > 2σ(*F* ^2^)] = 0.019
*wR*(*F* ^2^) = 0.052
*S* = 1.07
1844 reflections
71 parameters
H-atom parameters constrained
Δρ_max_ = 0.61 e Å^−3^
Δρ_min_ = −0.33 e Å^−3^

### 

Data collection: *APEX2* (Bruker, 2004 ▶); cell refinement: *APEX2* and *SAINT* (Bruker, 2004 ▶); data reduction: *SAINT* and *XPREP* (Bruker, 2004 ▶); program(s) used to solve structure: *SHELXS97* (Sheldrick, 2008 ▶); program(s) used to refine structure: *SHELXL97* (Sheldrick, 2008 ▶); molecular graphics: *ORTEP-3 for Windows* (Farrugia (1997 ▶); software used to prepare material for publication: *SHELXL97* and *PLATON* (Spek, 2009 ▶).

## Supplementary Material

Crystal structure: contains datablock(s) global, I. DOI: 10.1107/S1600536812033193/tk5133sup1.cif

Structure factors: contains datablock(s) I. DOI: 10.1107/S1600536812033193/tk5133Isup2.hkl

Additional supplementary materials:  crystallographic information; 3D view; checkCIF report

## Figures and Tables

**Table 1:** Hydrogen-bond geometry (Å, °)

*D*—H⋯*A*	*D*—H	H⋯*A*	*D*⋯*A*	*D*—H⋯*A*
N2—H2*B*⋯N1	0.86	2.26	3.079 (3)	159
N2—H2*A*⋯S1^i^	0.86	2.70	3.461 (3)	148
N3—H3*A*⋯S1^ii^	0.86	2.78	3.483 (3)	140
N3—H3*B*⋯S2^iii^	0.86	2.62	3.456 (3)	166

Symmetry codes: (i) 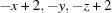; (ii) 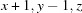; (iii) 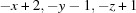.

**Fig. 1.**
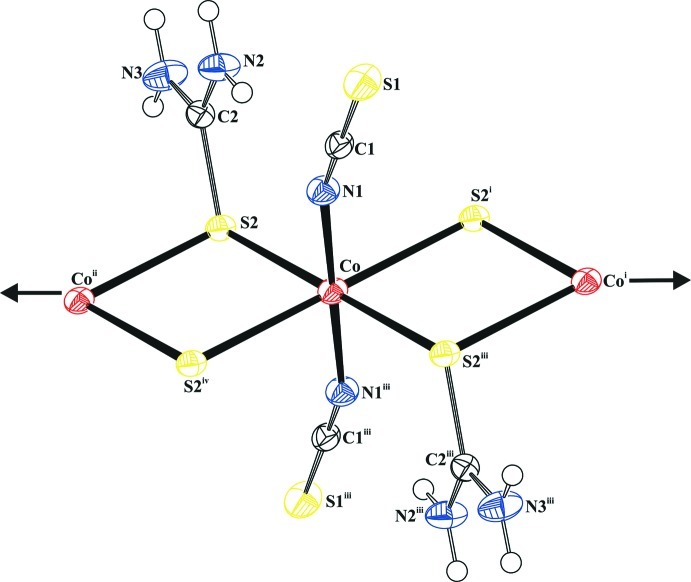
Asymmetric unit of the title complex expanded to show the coordination geometry of the CoII atom and the polymeric connectivity. Displacement ellipsoids are shown at the 50% probability level. H atoms are presented as a small circles of arbitrary radius. [Symmetry codes: (i)-1 + x, y, z; (ii)1 + x, y, z; (iii)1 - x, -y, 1 - z; (iv)2 - x, -y, 1 - z].

**Fig. 2.**
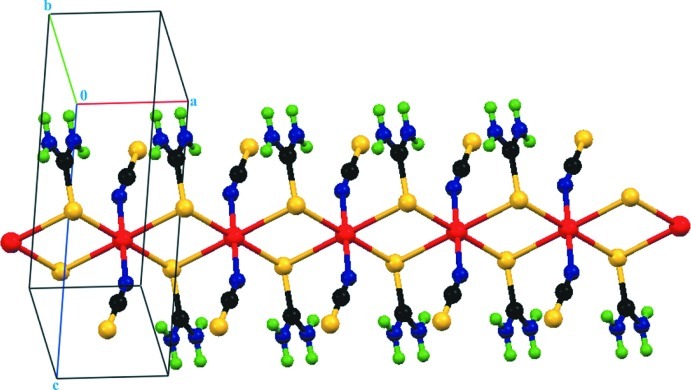
A view of the linear polymeric chain aligned along [100] in the title complex. Colour code: Co, red; N, blue; S, yellow; C, black; H, green.

## supplementary crystallographic information

### Comment

The interest in the coordination compounds possessing both thiourea and thiocyanato ligands dates back to the 1950's (*e.g.* Nardelli *et al.*, 1957) when the nature of coordination compounds formed by divalent cations (*M* = Mn, Co, Ni, Cd, Pb) and organic molecules containing sulfur was extensively studied. The interest in these compounds is related either to their non-linear optical properties (Yuan *et al.*, 1997, Yu *et al.*, 2001) or with their possible use as single-source precursors of semiconducting materials. Moreover, the use of SCN ligands, with bridging abilities, may lead to intriguing architectures and topologies, often generating one-dimensional chains (Machura *et al.*, 2011). For the above reasons and during our studies on new molecular precursors (Kropidłowska *et al.*, 2008), we turned our attention to systems of this type, that is, complexes containing thiourea and thiocyanate ligands connected to a cobalt center.

The title complex, Fig. 1, is isostructural with the previously reported cadmium(II) complex (Wang *et al.*, 2002). The Co^II^ atom is located at the inversion centre and is octahedrally coordinated by two N atoms from two thiocynate groups and four S atoms from four thiourea molecules. The bridging thiourea ligands link Co^II^ ions into a one dimensional polymeric chain along [100] (Fig. 2). The Co···Co distance along the chain is 3.855 (3) Å. The octahedral coordination sphere of the cobalt(II) cation is slightly distorted with distances in the range of 2.016 (1) Å to 2.623 (1) Å. The angles around the cobalt(II) atom range from 83.4 (1)° to 180°. The thiocynate group is almost linear with the N1—C1—S1 angle = 179.2 (1)°.

The molecular conformation is stabilized by intramolecular N2—H2B···N1 hydrogen bond, forming an *S*(6) ring motif (Bernstein *et al.*, 1995). In the crystal, molecules are linked by N—H···S hydrogen bonds into a three-dimensional architecture (Table 1).

### Experimental

Cobalt(II) chloride, ammonium thiocynate and thiourea were dissolved in aqueous solution in the molar ratio 1:2:2 and stirred well for 2 h to obtain an homogeneous mixture. The dark-brown crystals of the title compound were obtained after the filtrate and had been allowed to stand at room temperature for two weeks.

### Refinement

H atoms were positioned geometrically, with N—H = 0.86 Å and constrained to ride on their parent atom, with *U*_iso_(H)=1.2*U*_eq_(N).

### Crystal data

**Table:**

[Co(NCS)_2_(CH_4_N_2_S)_2_]	*Z* = 1
*M**_r_* = 327.33	*F*(000) = 165
Triclinic, *P*1	*D*_x_ = 1.925 Mg m^−^^3^
Hall symbol: -P 1	Mo *K*α radiation, λ = 0.71073 Å
*a* = 3.855 (3) Å	Cell parameters from 2298 reflections
*b* = 7.585 (2) Å	θ = 2.0–34.1°
*c* = 10.094 (2) Å	µ = 2.23 mm^−^^1^
α = 92.424 (3)°	*T* = 293 K
β = 98.172 (2)°	Block, brown
γ = 104.166 (2)°	0.24 × 0.22 × 0.16 mm
*V* = 282.4 (2) Å^3^	

### Data collection

**Table:**

Bruker APEXII CCD diffractometer	1844 independent reflections
Radiation source: fine-focus sealed tube	1764 reflections with *I* > 2σ(*I*)
Graphite monochromator	*R*_int_ = 0.026
Detector resolution: 10.0 pixels mm^-1^	θ_max_ = 34.1°, θ_min_ = 2.0°
ω scans	*h* = −5→5
Absorption correction: multi-scan (*SADABS*; Sheldrick, 1996)	*k* = −11→10
*T*_min_ = 0.591, *T*_max_ = 0.699	*l* = −14→15
6452 measured reflections	

### Refinement

**Table:**

Refinement on *F*^2^	Secondary atom site location: difference Fourier map
Least-squares matrix: full	Hydrogen site location: inferred from neighbouring sites
*R*[*F*^2^ > 2σ(*F*^2^)] = 0.019	H-atom parameters constrained
*wR*(*F*^2^) = 0.052	*w* = 1/[σ^2^(*F*_o_^2^) + (0.0264*P*)^2^ + 0.0757*P*] where *P* = (*F*_o_^2^ + 2*F*_c_^2^)/3
*S* = 1.07	(Δ/σ)_max_ = 0.001
1844 reflections	Δρ_max_ = 0.61 e Å^−^^3^
71 parameters	Δρ_min_ = −0.33 e Å^−^^3^
0 restraints	Extinction correction: *SHELXL97* (Sheldrick, 2008), Fc^*^=kFc[1+0.001xFc^2^λ^3^/sin(2θ)]^-1/4^
Primary atom site location: structure-invariant direct methods	Extinction coefficient: 0.203 (7)

### Special details

**Table:**

Geometry. All e.s.d.'s (except the e.s.d. in the dihedral angle between two l.s. planes) are estimated using the full covariance matrix. The cell e.s.d.'s are taken into account individually in the estimation of e.s.d.'s in distances, angles and torsion angles; correlations between e.s.d.'s in cell parameters are only used when they are defined by crystal symmetry. An approximate (isotropic) treatment of cell e.s.d.'s is used for estimating e.s.d.'s involving l.s. planes.
Refinement. Refinement of *F*^2^ against ALL reflections. The weighted *R*-factor *wR* and goodness of fit *S* are based on *F*^2^, conventional *R*-factors *R* are based on *F*, with *F* set to zero for negative *F*^2^. The threshold expression of *F*^2^ > σ(*F*^2^) is used only for calculating *R*-factors(gt) *etc*. and is not relevant to the choice of reflections for refinement. *R*-factors based on *F*^2^ are statistically about twice as large as those based on *F*, and *R*- factors based on ALL data will be even larger.

### Fractional atomic coordinates and isotropic or equivalent isotropic displacement parameters (Å^2^)

**Table:**

	*x*	*y*	*z*	*U*_iso_*/*U*_eq_	
C1	0.5577 (3)	0.18192 (15)	0.78972 (10)	0.01952 (19)	
C2	0.9805 (3)	−0.29358 (15)	0.69572 (11)	0.02016 (19)	
N1	0.5889 (3)	0.10262 (13)	0.69282 (9)	0.02280 (18)	
N2	0.9447 (3)	−0.19551 (16)	0.80075 (10)	0.0295 (2)	
H2A	0.9899	−0.2301	0.8800	0.035*	
H2B	0.8759	−0.0966	0.7903	0.035*	
N3	1.0858 (3)	−0.44519 (15)	0.71126 (11)	0.0324 (2)	
H3A	1.1313	−0.4801	0.7904	0.039*	
H3B	1.1091	−0.5092	0.6423	0.039*	
S1	0.51233 (9)	0.29477 (5)	0.92362 (3)	0.03241 (9)	
S2	0.89332 (7)	−0.22779 (3)	0.53449 (2)	0.01878 (8)	
Co	0.5000	0.0000	0.5000	0.01874 (8)	

### Atomic displacement parameters (Å^2^)

**Table:**

	*U*^11^	*U*^22^	*U*^33^	*U*^12^	*U*^13^	*U*^23^
C1	0.0193 (4)	0.0231 (5)	0.0165 (4)	0.0073 (4)	0.0011 (3)	0.0004 (3)
C2	0.0189 (4)	0.0209 (5)	0.0200 (4)	0.0033 (4)	0.0031 (3)	0.0046 (4)
N1	0.0259 (4)	0.0256 (4)	0.0169 (4)	0.0078 (4)	0.0022 (3)	−0.0016 (3)
N2	0.0430 (6)	0.0313 (5)	0.0173 (4)	0.0147 (4)	0.0056 (4)	0.0040 (4)
N3	0.0484 (6)	0.0269 (5)	0.0256 (5)	0.0173 (5)	0.0027 (4)	0.0072 (4)
S1	0.03586 (17)	0.0469 (2)	0.01841 (14)	0.02104 (14)	0.00223 (11)	−0.00877 (12)
S2	0.02254 (13)	0.01991 (13)	0.01566 (12)	0.00861 (9)	0.00307 (9)	0.00202 (8)
Co	0.02202 (11)	0.02291 (12)	0.01230 (10)	0.00862 (8)	0.00200 (7)	−0.00215 (7)

### Geometric parameters (Å, º)

**Table:**

C1—N1	1.1627 (14)	N3—H3A	0.8600
C1—S1	1.6226 (11)	N3—H3B	0.8600
C2—N2	1.3121 (15)	S2—Co	2.5668 (10)
C2—N3	1.3182 (15)	S2—Co^i^	2.6231 (14)
C2—S2	1.7338 (11)	Co—N1^ii^	2.0158 (10)
N1—Co	2.0158 (10)	Co—S2^ii^	2.5668 (10)
N2—H2A	0.8600	Co—S2^iii^	2.6231 (14)
N2—H2B	0.8600	Co—S2^iv^	2.6231 (14)
			
N1—C1—S1	179.17 (10)	N1—Co—S2^ii^	83.37 (3)
N2—C2—N3	120.18 (11)	N1^ii^—Co—S2^ii^	96.63 (3)
N2—C2—S2	121.31 (9)	N1—Co—S2	96.63 (3)
N3—C2—S2	118.50 (9)	N1^ii^—Co—S2	83.37 (3)
C1—N1—Co	160.34 (9)	S2^ii^—Co—S2	180.000 (11)
C2—N2—H2A	120.0	N1—Co—S2^iii^	88.73 (3)
C2—N2—H2B	120.0	N1^ii^—Co—S2^iii^	91.27 (3)
H2A—N2—H2B	120.0	S2^ii^—Co—S2^iii^	95.93 (5)
C2—N3—H3A	120.0	S2—Co—S2^iii^	84.07 (5)
C2—N3—H3B	120.0	N1—Co—S2^iv^	91.27 (3)
H3A—N3—H3B	120.0	N1^ii^—Co—S2^iv^	88.73 (3)
C2—S2—Co	117.06 (4)	S2^ii^—Co—S2^iv^	84.07 (5)
C2—S2—Co^i^	104.64 (4)	S2—Co—S2^iv^	95.93 (5)
Co—S2—Co^i^	95.93 (5)	S2^iii^—Co—S2^iv^	180.000 (11)
N1—Co—N1^ii^	180.0		
			
S1—C1—N1—Co	−33 (7)	C2—S2—Co—N1	21.76 (5)
N2—C2—S2—Co	−19.76 (11)	Co^i^—S2—Co—N1	−88.02 (3)
N3—C2—S2—Co	160.47 (8)	C2—S2—Co—N1^ii^	−158.24 (5)
N2—C2—S2—Co^i^	84.92 (10)	Co^i^—S2—Co—N1^ii^	91.98 (3)
N3—C2—S2—Co^i^	−94.85 (10)	C2—S2—Co—S2^ii^	−117 (100)
C1—N1—Co—N1^ii^	−140 (100)	Co^i^—S2—Co—S2^ii^	133 (100)
C1—N1—Co—S2^ii^	12.8 (3)	C2—S2—Co—S2^iii^	109.79 (4)
C1—N1—Co—S2	−167.2 (3)	Co^i^—S2—Co—S2^iii^	0.0
C1—N1—Co—S2^iii^	108.9 (3)	C2—S2—Co—S2^iv^	−70.21 (4)
C1—N1—Co—S2^iv^	−71.1 (3)	Co^i^—S2—Co—S2^iv^	180.0

Symmetry codes: (i) *x*+1, *y*, *z*; (ii) −*x*+1, −*y*, −*z*+1; (iii) −*x*+2, −*y*, −*z*+1; (iv) *x*−1, *y*, *z*.

### Hydrogen-bond geometry (Å, º)

**Table:**

*D*—H···*A*	*D*—H	H···*A*	*D*···*A*	*D*—H···*A*
N2—H2*B*···N1	0.86	2.26	3.079 (3)	159
N2—H2*A*···S1^v^	0.86	2.70	3.461 (3)	148
N3—H3*A*···S1^vi^	0.86	2.78	3.483 (3)	140
N3—H3*B*···S2^vii^	0.86	2.62	3.456 (3)	166

Symmetry codes: (v) −*x*+2, −*y*, −*z*+2; (vi) *x*+1, *y*−1, *z*; (vii) −*x*+2, −*y*−1, −*z*+1.
